# Consumer Preference and Quality of Sachet Water Sold and Consumed in the Sunyani Municipality of Ghana

**DOI:** 10.1155/2020/3865895

**Published:** 2020-08-05

**Authors:** Henry Ofosu Addo, Kingsley Ebenezer Amegah, Thelma Arko Xonam, Eunice Kabenlah, Charity Ameyaw, Linda Obema Graham, Theresa Tobigah, Barbara Hammond, Dorcas Maxwell, Langbong Bimi

**Affiliations:** ^1^Department of Animal Biology and Conservation Science, School of Biological Sciences, University of Ghana, Legon, Ghana; ^2^Centre for Plant Medicine Research, Mampong-Akuapem, Ghana; ^3^Department of Health Information, Hohoe Municipal Hospital, Hohoe V/R, Ghana; ^4^Faculty of Public Health and Allied Sciences, Catholic University College of Ghana, Ghana; ^5^Institute of Environment and Sanitation Studies, University of Ghana, Legon, Ghana

## Abstract

Good quality water is odourless, colourless, and free from faecal pollution, and a satisfactory safe supply must be made available to consumers. The study assessed consumer preference and quality of sachet water sold and consumed in the Sunyani Municipality of Ghana. A cross-sectional study design was used, and data were collected using a structured questionnaire from a sample size of 500 respondents. A total of twenty (20) samples of sachet water commonly sold and consumed in the Sunyani Municipality were also collected and analyzed for bacteriological and physicochemical parameters. Out of these 20 samples, 10 samples of sachet water were collected from the production site and the other 10 samples from the market site. Results showed that respondents' choice of sachet water was based on several indicators. While 70% (350/500) of consumers indicated that sachet water had taste, 58% (290/500) of them were not enthused when the water had colour. Using smell as an indicator, 71% of consumers have had an experience with sachet water smell being questionable. Water taste (*p* < 0.001), colour of water (*p* < 0.001), smell (*p* < 0.001), and increase in cost (*p* < 0.001) were found as determinants of consumer preference. Consumers who said the colour of water informed their decision when buying sachet water were seven times more likely to have a preference for a particular brand (OR 7.057, CI: 4.474–11.133). Those who checked for expiry dates when buying water (OR 4.871, CI: 3.110–7.628) and those who looked out for Food and Drugs Authority stamp (OR 4.433, CI: 2.806–7.003) were both four times more likely to have a preference for a particular brand. Water quality tests also indicated that 30% of all water samples collected from the production site were contaminated with total coliforms and 50% of sachet water samples collected from the markets were also contaminated with coliforms. The same brands of sachet water that contained total coliforms from the production site were the same brands that contained total coliforms selected from the market. From this study, only total coliforms other than *Escherichia coli* were detected in the water samples analyzed. It was observed that the evaluated physicochemical parameters of the water analyzed were within the accepted WHO limits. It is recommended that consumers be educated about the indicators to look out for when buying sachet water and, also, that regulatory bodies be empowered to ban the sale of unwholesome brands of sachet water on the market.

## 1. Introduction

Drinking water of good quality is required to sustain life. Access to water is thus noted as a fundamental human right more so in developing countries where access to potable water is lacking. The sale and consumption of packaged water are growing increasingly in West Africa and in other middle and low income countries of the world [1].

Access to and consumption of sachet water have increased in Ghana over the last few years. Thus, the production and sale of sachet water have become a booming business venture in Ghana, with so many brands of sachet water on the market. Globally, the sale of sachet water has also increased in recent years due to the perception that it is safe, hygienic, and/or handy and also because of its aesthetic appeal (Doria, Pidgeon, & Hunter, [2, 3]). The brands are so many that one tends to question whether these production companies do really adhere to all safety protocols in their production.

The water vending industry in Ghana has gone through many transformations. Initially, hawkers carried water in containers on their heads and sold to consumers from plastic cups. This raised a lot of safety and health issues as different consumers shared the same cup. Then came water sold in hand-tied plastic bags. This also raised several questions especially about the source of the water which was usually of dubious quality and also hygienic practices especially in the handling. Currently, sachet water consists of 500 ml of plastic bags of water that are heat sealed [4] and popularly termed as “*pure water*” due to its perceived safe and hygienic condition.

**Figure 1:**
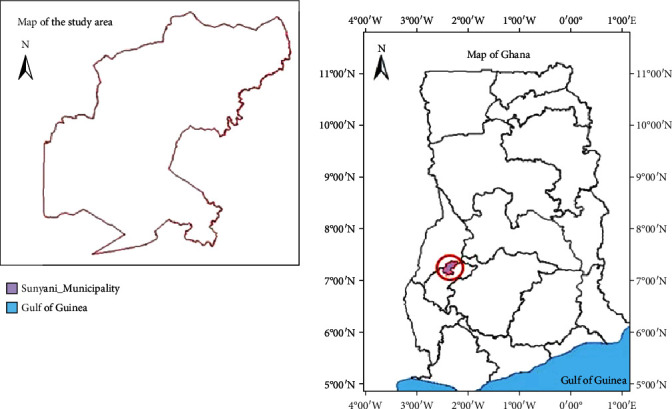
Map of Ghana showing Sunyani Municipality.

Sachet water is highly patronized by most people in the Sunyani Municipality of Ghana. This high patronage could lead to substandard products on the market because of profit (WHO Fact Sheet, 2000). Sachet water in the Sunyani Municipality can be obtained from three sites: production site, vending site, and hawkers. Although, as much as 29% of Ghanaians depend on sachet water for various purposes, lack and/or inadequate access to water means that most households depend on other sources of water for daily activities like washing and bathing, while sachet waters are mostly drunk (Ghana Demographic and Health Survey, 2014). The Ghana Demographic and Health (2014) Survey revealed that 43% of urban dwellers and 11.8% of rural dwellers, respectively, rely on sachet water. This need therefore has led to the proliferation of various sachet water-producing companies in the Sunyani Municipality, most of which are unregistered and not certified by the relevant regulatory agencies. There are so many brands of sachet water clamoring for the attention of the unsuspecting consumer. The question then is this: What does the consumer look out for before purchasing a particular brand of sachet water? What are the consumer indicators that influence consumers' preference for a particular brand of water? Quality water should be colourless, tasteless, odourless, and free from faecal contamination [5]. Access, hygiene, purity, taste, cost, and most importantly safety are among various reasons for sachet water consumption [6]. Previous studies conducted bacteriological analysis of sachet water carried out in some parts of Ghana which were shown to be contaminated [7, 8]. Sunyani Municipality is an urbanized area in the transitional belt of Ghana (Figure 1) where several brands of sachet water are sold to the public.

Most studies have only looked at the microbial and chemical quality of sachet water and the possible health outcomes of drinking unsafe water [9, 10]. Yet no study has been done in the Sunyani Municipality on the factors that play major roles in the choices of consumers on the brand of sachet water they consume despite the large number of the population depending on sachet water for their daily consumption and whether they trust the brand they consume.

This study therefore looked at consumer indicators that influence their preference for a particular brand of sachet water and the bacteriological and physicochemical quality of sachet water sold and consumed in the Sunyani Municipality.

## 2. Materials and Methods

### 2.1. Study Area

Sunyani Municipality is the administrative capital of the Bono Region of Ghana. It lies between Latitudes 7°20′N and 7°05′N and Longitudes 2°30′W and 2°10′W and shares boundaries with Sunyani West District to the North, Dormaa District to the West, Asutifi District to the South, and Tano North District to the East. In 2000, the population of Sunyani municipality was 101,145. Currently, with a growth rate of 3.8 percent, the estimated population is 147,301. The high densely populated areas in the municipality include Zongo, New Dormaa, and Area 2 in that order. The low-density areas are Estate, South Ridge, Airport Area, Atronie, and Baakoniaba. The study was conducted in both the high and low densely populated areas.

### 2.2. Study Design and Sampling Procedure

A descriptive cross-sectional study design was adopted, and cluster sampling technique was used in selecting towns within Sunyani Municipality for the study (Figure 1). The Municipality was clustered into high densely populated areas and low densely populated areas. Out of these clusters, simple random sampling technique was used to select two subdistricts from the high densely populated areas (Zongo and New Dormaa) and another two subdistricts from the low densely populated areas (Atronie and Baakoniaba). From these four subdistricts, convenience sampling technique was used to select 500 consumers of sachet water including vendors and hawkers for the study.

#### 2.2.1. Sample Size

The sample size was determined using single population formula: *N* = (*Z*^2^ × *p*(1 − *p*))/*d*^2^, at 95% confidence interval and 5% margin error (*d*) with assumed quality of water (*p*) of 50%, where *N* is the sample size, *Z*^2^ (statistic) = 1.96, *p* (quality of water) = 0.5, and *d* (margin of error) = 0.05: *N* = (1.96^2^ × 0.5(1 − 0.5))/0.05 = 384.16. The sample size after adjusting for 10% was 424 study participants. To account for design, the sample size was adjusted to 500 respondents for this study.

A purposive sampling technique was used to target ten (10) producers of sachet water. This sachet water producer list was obtained from the Environmental Health Department. Vendors of sachet water for this study were defined as immobile sachet water sellers, and hawkers for this study were defined as individuals who are mobile and go round the town selling sachet water. These vendors and hawkers are concentrated around the Sunyani station and market. To select these vendors and hawkers, convenience sampling techniques were used. All consenting vendors and hawkers at the time of the study were recruited to be part of the study. To select consumers of sachet water for this study, various sachet water hawkers were followed and convenience sampling technique was used to select all those who orally consented to be part of the study (Table 1).

### 2.3. Data Collection Procedure

A structured questionnaire was used to collect data from the respondents. Data was collected between January 2018 and April 2018 by trained research assistants recruited by the authors. These research assistants were undergraduate students of the Catholic University College of Ghana. The questionnaires were filled out while interacting with consumers. The questionnaires consisted of both open and closed ended questions. Sections from the questionnaires included demographic data, access and use of sachet water, and role played by regulatory bodies. To address the preference of consumers of sachet water, various consumer indicators such as taste, colour, odour, cost, and labels on sachet water were assessed. A total of 20 brands of sachet water were randomly selected from both the production site and the market. Two replicate samples were collected per brand. The samples were taken to the Ghana Water Company Laboratory in Sunyani in insulated containers with ice parks to prevent contamination of the samples collected. Analyses were carried out within 8 hours after collection. Where immediate microbiological evaluation was going to be delayed, the samples were refrigerated at 4°C and analyzed within 24 hours of collection. This prevented the contamination of the samples collected.

#### 2.3.1. Bacteriological Quality Determination

(1) Total Coliform

This was determined by the Most Probable Number (MPN) index method using 5-5-5 regimen. MacConkey broth was used in determining total coliform. Single strength MacConkey broth was prepared, and 40 g to 1 litre of distilled water was distributed into containers filtered with Durham's tube sterilized by autoclaving at 121°C for 15 minutes.

(2) Faecal Coliform

This was determined by using Brilliant Green. Forty (40) g of Brilliant Green powder was weighed and dispersed it into 1 litre of deionized water and allowed to soak for 10 minutes and dispensed into tubes or bottles with inverted Durham's tubes and sterilized by autoclaving at 115°C for 15 minutes and incubated at 37°C for 24 or 48 hours and if colour changes from pink or purple to yellow with a gas in Durham's tube which indicated faecal coliform.

(3) Escherichia coli

Peptone water was used to determine the presence of *Escherichia coli* (*E. coli*). Suspended 15 g of peptone water was placed into 100 ml of distilled water and was dispersed into tubes with or without Durham's tube and sterilized by autoclaving at 151 bs pressure (121°C) for 15 minutes and incubated at 37°C for 24 hours and if the colour changes from pink or purple to yellow with a gas in Durham's tube which indicate faecal coliform.

#### 2.3.2. Physicochemical Parameters

(1) pH

The pH readings of the water samples were taken using pH meter HACH HQ11D. Instruments were calibrated by using pH buffer solutions 4.7 and 10.

(2) Colour

A platinum-cobalt standard method was used. Filters were rinsed by pouring 50 ml of deionized water through the filter. Blank sample cells were filled with 25 ml deionized water, and excess was discarded. Stored programmed number 120 was entered, and it displayed 455 on the HACH HQ11D Instrument. Wavelength was rotated until it displaced 455 nm. Fifty (50) ml of sample was poured through the filter. The prepared sample was filled with 25 ml of filtered sample and placed into a cell holder, and the light shield was closed and the response in platinum-cobalt was shown.

(3) Alkalinity

Titrant used was 0.02 m HCl into 0.5 ml of methyl orange solution added into 100 ml of sample. The colour changed from red to orange. The result titrants were multiplied by 10 units (mg/l).

(4) Total Hardness

1 m of ammonia buffer and 0.5 m of eriochrome black T were put into 100 ml of sample. The colour changed from pink to light blue or green. The result titrants were multiplied by 10 units (mg/l).

(5) Chloride

Titrant used was 0.02 m silver nitrate. The indicator used was 1 ml of potassium chromate solution which was put into 100 ml of sample, and the colour changed from yellow to brown. The result titrants were multiplied by 10 units (mg/l).

(6) Iron

Stored programme number 265 was entered and displayed 510 nm; wavelength was rotated until it displayed 510 nm. The displayed showed mg/l Fe, and a cell was filled with a sample. The contents of one FerroVer Iron Regent Powder were added to the sample cell (the prepared sample was swirled to mix).

(7) Nitrate

The stored programme number for high range nitrate nitrogen (NO_3_-N) powder pillows 355 reader was pressed until it showed 500. A sample cell was filled with 25 ml of sample, one content of nitrate powder pillow was added to the cell, and the prepared sample was swirled to mix. Another sample cell was filled with 25 ml of sample. The stopper was removed, and the prepared sample was placed into the cell holder. The displayed showed wait and then resulted in mg/l nitrate nitrogen (NO_3_-N).

(8) Phosphate

PhosVer 3 Phosphate powder pillow was used. The sample cell was filled with 25 ml of sample. The contents of one PhosVer 3 Phosphate powder pillow were added to the cell and swirled immediately to mix. Another sample cell was filled with 25 ml of sample and placed into the cell holder. The timer beeps to display mg/l PV. After 30 minutes, the sample was placed into the cell holder, and the light shield was closed. The result in mg/l was displayed.

### 2.4. Data Analysis

The data entry and analysis were performed using the Statistical Package for Social Sciences (SPSS version 20) processing software. Univariate analysis was used to generate both absolute and relative frequencies. Bivariate analysis (*chi-square test*) was also used to test association between the outcome or dependent variable (preference of sachet water) and independent variables. The independent variables used in this study included educational level, occupation, age, sex, and specific brand of sachet water purchased as consumer preferences are influenced by these. The analysis further included the calculation of odds ratios to test the strength of association between independent variables and the preference of sachet water through a multiple logistic regression analysis. Preference of sachet water was measured based on various indicators outlined by consumers which were colour, taste, odour, and cost.

Logistic regression analysis was used for this study because logistic regression studies the association between a dependent variable (preference for sachet water) which is dichotomous and a set of independent or explanatory variables. The main objective of logistic regression is to find the best fitting model to describe the relationship between the dependent and the independent variables of interest. Thus, to explain the relationship between the preference for sachet water and the independent variables, a binary logistic regression model was fitted to examine whether the presence of authority stamp, address of producers, colour, smell, cost, brand, and expiry date of water statistically and significantly impact the odds of preference for sachet water. Univariate logistic regression was first conducted to test the influence of sachet water preference and sachet water indicators such as taste, smell, cost, brand, expiry date, and address of producers. Considering *p* < 0.05 as significant, all significant variables were put into a multiple logistic regression model to calculate adjusted odds ratio. This was done to control for confounders, and as such, odds ratio was used instead of a prevalence ratio which mainly assesses the proportion of persons with disease over the proportion with exposure.

### 2.5. Ethical Considerations

Ethical clearance was sought from the Ethics Committee of the Sunyani Municipal Health Directorate, the Municipal Assembly, Food and Drugs Authorities, and from the various representatives of the subdistricts. The participants were also duly informed that their participation in the study was purely voluntary, and as such, they could choose to partake or not. That is, the administration and completion of the questionnaires were without any form of coercion, and handling of unresponsive interviewees during the process was also explained. All those who orally consented were thus recruited to be part of the study. Confidentiality of their responses however was of high priority and was not compromised in any way.

## 3. Results

### 3.1. Background Characteristics of Respondents

The study consisted of 500 consumers of sachet water. This included hawkers, food vendors, producers, and other consumers. From the results, most (52%) consumers were females and less than half (46%) of them were within ages 26 to 35 years. A quarter of them were below age 25 years with only 16% being from ages 36 to 45 (see Table 2).

### 3.2. Consumer Indicators and Sachet Water

The study revealed that about seven in every ten consumers have the belief that sachet water has taste. Among this group, 78% said the taste of a sachet water informs their decision to buy a particular brand of water. Fifty-eight percent (58%) were of the view that water has colour; only 61% made a decision to buy a particular brand of sachet water due to its colour. On the side of smell as an indicator, 71% of consumers have had an experience with sachet water smell being questionable. The cost of sachet water was also another indicator of relevance to consumers. Fifty-five percent (55%) of the respondents said their decision to buy a particular brand of water depended on the monetary value of that brand. Surprisingly, 78% of consumers do not check for food and drugs board labels on sachet water in making their preference, and 76% of them do not check expiry dates before buying or consuming sachet water.

### 3.3. Relationship between Consumer Indicators and Preference of Sachet Water

A bivariate analysis was conducted to ascertain the association between the outcome variable (Preference of sachet water) and various independent variables. The results indicated that consumers believed that water has taste (*p* < 0.001), and decisions made as a result of this belief (*p* < 0.001) were both associated with their preference for a particular brand sachet water. The statistics further indicated that respondents who make decisions on the brand of water to buy due to colour (*p* < 0.001) and those who used their preferred brand of sachet water because of its colour (*p* < 0.001) were likely to have a preference for a particular brand of sachet water. Consumer awareness that water has a smell (*p* < 0.001) and whether they ever experienced water with a particular smell (*p* < 0.001) were all associated with their preference. Consumer indicators such as cost and checking of labels were all statistically related to the preference of a particular brand of sachet water (*p* < 0.001) (Table 3).

### 3.4. Determinants of Consumer Preference of Sachet Water

In a multiple logistic regression model, adjusted odds ratios were calculated to control for confounders. After controlling for confounders, taste (OR 0.005, CI: 0.002–0.012), colour of water (OR 0.089, CI: 0.053–0.148), the smell of the water (OR 0.129, CI: 0.082–0.203), the cost of a particular brand (OR 0.258, CI: 0.258–0.399), and increase in cost (OR 0.210, CI: 0.136–0.323) were found as determinants of consumer preference.

However, consumers who said the colour of water informed their decision when buying sachet water were seven times more likely to have a preference for a particular brand (OR 7.057, CI: 4.474–11.133). Those who checked for expiry dates when buying water (OR 4.871, CI: 3.110–7.628) and those who look out for Food and Drugs authority stamp on the water before making a decision to buy a particular brand of sachet water (OR 4.433, CI: 2.806–7.003) were both four times more likely to have a preference. Even those who checked the address of producers before buying also had higher odds (OR 3.884, CI: 2.376–6.348) (Table 2).

## 4. Discussion

Access to water still remains a challenge in most developing countries including some communities in Ghana. Some communities in Ghana rely on other sources of water for their domestic activity like washing and bathing but resort to sachet water for cooking and drinking purposes. This study looked at what informed consumers on the brand of sachet water they purchased and used and its quality. The availability of drinking water that is not only safe in terms of its quality but also acceptable in its colour and odour is of utmost priority to the consumer. Water that is meant for drinking purposes that has good aesthetic value will appeal to the confidence of the consumer.

Concerning whether the colour of water played any role in their preference of a particular brand of water, 290 respondents (58%) affirmed that water has colour (Table 5). Water of good quality should not have colour. This goes contrary to the study of Oludairo & Aiyedun [5] who opined that water should be colourless. It was found that colour as an indicator was significant in informing consumers in making their preference of sachet water (*p* < 0.05). Previous findings have also indicated similarity with this current finding [11]. In addition, consumers who said the colour of water informed their decision when buying sachet water were seven times more likely to have a preference for a particular brand (OR 7.057, CI: 4.474–11.133). This means that consumers of sachet water were aware of the implications of drinking water that had colour as the colour rendered the water impure for human consumption.

Out of 500 respondents, 325 (65%) observed that in one way or the other water has odour or smell, which contradicts Oludairo & Aiyedun [5] which report water should be odourless. In a cross-sectional study done in South Africa, similar results indicate perceived drinking water safety and preference are primarily related to water taste, as perceived by respondents [12], and general acceptability of drinking water depends on odour [13]. In probing about the smell of water, majority (71%) indicated that indeed water can have odour or smell but will be due to some substances from the source and also how the water is stored. It was found that the smell or odour made consumers opt for a particular brand to another brand (*p* < 0.05) as similarly found by [14]. However, the smell of sachet water was less likely to determine preference of sachet water by consumers (OR 0.129, CI: 0.082–0.203).

The findings of the study revealed that 75% of the respondents believed water has taste and this contradicts the study of [5]. On the other hand, there was a relationship between taste and odour as an indicator that informed consumers to choose a particular brand of sachet water. Questionable taste or odour could lead the consumer to believe that the water is unsafe and may prefer a different brand of water [15]. Although the taste of water informed consumers to choose a particular brand of sachet water, they were however less likely to make a preference for another brand of sachet water due to its taste (OR 0.006, CI: 0.003–0.013). In other national surveys, however, conscious patronage of brands exhibiting better taste and no particles was used as a quality control preference among both consumers and producers [16]. The presence of odour in drinking water could be attributable to metal concentrations, organisms, and chemicals.

The present study ascertained the influence of cost on the preference of sachet water, and it was found that 345 respondents representing 69% agreed that an increase in cost will influence their choice of sachet water. As stated in similar views, sachet water with low cost price readily became an available alternative for water provision and choice of water [13]. Since there is an increase, consumers' preference for a particular brand has changed in the way that some vendors and hawkers sell at the new price while others sell at the old price. This means that if one consumer's preferred brand has been increased in price and another brand is still sold at the old price, the consumer will most likely move to buy the low-cost brand making his preference change due to increase in cost. Therefore, cost influenced consumers' preference for a particular brand of sachet water (*p* < 0.001). However, the likelihood of preferring a particular brand to another due to its cost was less (OR 0.258, CI: 0.258–0.399). In another study location, reduced cost price was similarly associated with purchase of water from manufacturers [17].

A sizeable number of sachet water brands in the country, especially those in the hinterlands, are produced, distributed, and consumed on the blind side of the Ghana Standards Authority (GSA) and the Food and Drugs Authority (FDA)—the institutions mandated to protect consumers against uncertified goods. Such brands are normally without the GSA and FDA labels. Consequently, previous literatures have suggested that all water that fails NAFDAC and WHO regulations should be retreated before they are released to the public for human consumption [18]. The said labels consist of the product, the brand name or trade name if any, the net volume, name and address of manufacturer, the batch code, and the expiry date indicated by the words “*Best Before*” [19]. Other requirements in Nigeria, however, included nutritional information, net volume [20], and NAFDAC registration number [17]. In the study, consumers were asked about the responsibilities of checking these labels. The certification of sachet water by these bodies gives consumers confidence about the safety and quality of the product they are consuming. From the results, 22% of respondents checked label for Foods and Drugs Authority endorsement before drinking sachet water. In another national study, 85% of consumers observed safety labels including expiring dates before consuming sachet water [21]. However, opposing results suggest that 78% of consumers in this study never checked for labels from these regulatory bodies. This is a worrying situation. Does this mean that consumers do not believe the work done by these authorized bodies? It was found that those who checked for expiry dates when buying water (OR 4.871, CI: 3.110–7.628) were four times more likely to have a preference. Contrary to this, another study in the Ashanti Region of Ghana suggests that nearly all (99%) of consumers never checked for expiring dates and indicated no choice of preference [22]. The study went further to assess whether consumers looked out for barcodes on the sachet water. About 87% of the consumers indicated that they had no idea about barcodes on the sachet water which are hence not important in their preference for a particular brand of sachet water. This is worrying because barcodes are very important for fast identification of a particular product. This means that consumers should be educated on the indicators they should look for before buying any brand of sachet water. From this study, consumers who checked for barcodes on sachet water were 3 times more likely to have a preference of sachet water (OR 3.699, CI: 2.172–6.298). This could be a novel finding in this study because there seems to be no literature available regarding consumer watch on barcodes of sachet water as an indicator of preference especially in the Sunyani Municipality. With respect to checking the address of producers, 83% did not check and 17% checked. In a similar consumer assessment, more than half (53%) of sachet water consumers never checked producer's address as a preference [22]. However, those who checked for address when buying water (OR 4.871, CI: 3.110–7.628) were four times more likely to have a preference. From the sachet water quality analyses (bacteriological and physicochemical), 30% of water samples selected from the production site were contaminated with total coliforms and 50% of samples selected from the market were also contaminated with coliforms. The worrying trend was that the same brands of sachet water that contained total coliforms from the production site were the same brands that contained total coliforms selected from the market. This shows poor water handling in most settings in Ghana regarding drinking water (Addo, Addo, & Bimi, [23]). No faecal coliforms and *E. coli* were detected in the water samples. From the study, the mean total coliforms ranged from 2.3 × 10^4^ to 4 × 10^4^ (Tables 6(a) and 6(b)). These values deviate from the World Health Organization's guidelines for drinking water quality. This states that for water intended for drinking, no total coliform should be detectable in 100 ml of water. All the values recorded for physicochemical parameters were within the World Health Organization's recommended limits (Table 7). This is consistent with the study by Dzodzomenyo et al. [24] where all sachet water tested were compliant with national quality standards.

## 5. Conclusions and Recommendations

There are many brands of sachet water on the market that confuse consumers as to which one they should go for. Majority of consumers have many indicators that influence their choice of sachet water. Any wrong choice on their part can have far reaching consequences such as health problems and even death. Factors which influence consumers in their preference of sachet water include taste, odour, colour, and cost. Consumers also look for certifications from appropriate bodies such as the Food and Drugs Authority (FDA) and Ghana Standards Authority (GSA) before purchasing a particular brand of sachet water. No *E. coli* was found in this study, and generally, all the water samples were within the World Health Organization's recommended values for physicochemical parameters. This study therefore recommends that proper education should be given to consumers especially on the importance of barcodes and other indicators found on the sachet water package. Also, regulatory bodies such as FDA and GSA should be empowered to ban sachet water products that do not meet their specifications.

## Figures and Tables

**Table 1:** List of respondents used for this study.

Participants	Frequency
Sachet water producer	10
Vendors	80
Hawkers	100
Consumers	310
Total	500

**Table 2:** Determinants of consumer preferences to sachet water (multiple logistic regression analysis) and sociodemographics.

Determinants	Preference of sachet water		
	AOR	95% CI	
Use of brand because of its taste			
Yes	.006	.003	.013
No	Ref		
Do you think water has colour			
Yes	.089	.053	.148
No	Ref		
Colour of sachet water informs decision			
Yes	7.057	4.474	11.133
No	Ref		
Still use preferred brand because of colour			
Yes	.114	.071	.181
No	Ref		
Water has smell			
Yes	.129	.082	.203
No	Ref		
Cost informs decision to buy			
Yes	.258	.258	.399
No	Ref		
Increase in prices informs decision to buy			
Yes	.210	.136	.323
No	Ref		
Check for expiry date			
Yes	4.871	3.110	7.628
No	Ref		
Realization of no bar codes on sachet water			
Yes	3.699	2.172	6.298
No	Ref		
Check address of producer			
Yes	3.884	2.376	6.348
No	Ref		
Sociodemographic characteristics	Preference of sachet water		
Sex	Yes, *n* (%)	No, *n* (%)	
Yes	186 (78.2)	52 (21.8)	
No	191 (72.9)	71 (27.1)	
Age			
15-25 years	121 (73.3)	44 (26.7)	
26-35 years	183 (79.6)	47 (20.4)	
36-45	58 (74.4)	20 (25.6)	
46-55	13 (56.5)	10 (43.5)	
Above 56 years	2 (50)	2 (50)	
Level of education			
Primary	78 (88.6)	10 (11.4)	
JHS	133 (84.2)	25 (15.8)	
SHS	62 (65.3)	33 (34.7)	
Tertiary	12 (26.1)	34 (73.9)	
Vocation	15 (60)	10 (40)	
No education	77 (87.5)	11 (12.5)	
Occupational status			
Government worker	10 (27.8)	26 (72.2)	
Private worker	42 (82.4)	9 (17.6)	
Self employed	165 (85.9)	27 (14.1)	
Trader	61 (82.4)	13 (17.6)	
Farmer	34 (81)	8 (19)	
Unemployed	40 (59.7)	27 (40.3)	
Student	25 (65.8)	13 (34.2)	

AOR = adjusted odds ratio.

**Table 3:** Relationship between consumer indicators and preference of sachet water.

Consumer indicators	Preference of sachet water		
	Yes, *n* (%)	No, *n* (%)	*p* value
Water has taste			
Yes	377 (100)	0 (0.0)	.001
No	0 (0.0)	123 (100)	
Taste informs you to buy			
Yes	368 (94.4)	22 (5.6)	.001
No	9 (8.2)	101 (91.8)	
Water has colour			
Yes	268 (92.4)	22 (7.6)	.001
No	109 (51.9)	101 (48.1)	
Colour informs decision to buy			
Yes	275 (89.0)	34 (11)	.001
No	102 (53.4)	89 (46.6)	
Water has smell			
Yes	290 (88.7)	37 (11.3)	.001
No	87 (50.3)	86 (49.7)	
Ever experience the smell of sachet water questionable			
Yes	296 (82.9)	61 (17.1)	.001
No	81 (56.6)	62 (43.4)	
Cost informs decision to buy			
Yes	239 (66.3)	38 (13.7)	.001
No	138 (61.9)	85 (38.1)	
Label of food and drug stamp affect decision			
Yes	55 (50.9)	53 (49.10)	.001
No	322 (82.1)	70 (17.9)	
Check of expiry date			
Yes	60 (50.4)	59 (49.6)	.001
No	317 (83.2)	64 (16.8)	
Realization of no barcodes			
Yes	34 (50.7)	33 (49.3)	.001
No	343 (79.2)	90 (20.8)	
Check address of producers before buying			
Yes	43 (51.2)	41 (48.8)	.001
No	334 (80.3)	82 (19.7)	

**Table 4:** Preference of sachet water.

Preference of sachet water	Frequency	Percent
Yes	377	75.4
No	123	24.6

From this study, a total of 377 people representing 75.4% preferred the usage of sachet water (Table 4).

**Table 5:** Consumer preference indicators of sachet water.

Variable	Category	Frequency	%
Water has taste	No	123	24.6
	Yes	377	75.4
	Total	500	100.0
Taste informs decision to buy	No	110	22.0
	Yes	390	78.0
	Total	500	100.0
Water has colour	No	210	42.0
	Yes	290	58.0
	Total	500	100.0
Water has smell	No	173	34.6
	Yes	327	65.4
	Total	500	100.0
Experience with water smell being questionable	No	143	28.6
	Yes	357	71.4
	Total	500	100.0
Increase in cost informs choice of sachet water	No	155	31.0
	Yes	345	69.0
	Total	500	100.0
Check of expiry date	Yes	119	23.8
	No	381	76.2
	Total	500	100.0
Realization of no barcodes	Yes	67	13.4
	No	433	86.6
	Total	500	100.0
Check address of manufacturer before buying	Yes	84	16.8
	No	416	83.2
	Total	500	100.0

**Table:** (a) Microbial quality of sachet water collected from production site

Samples of sachet water	Total coliform/100 ml	Faecal coliform/100 ml	*Escherichia coli*
Production site	Production site	Production site	Production site
Brand 1	Nil	Nil	Nil
Brand 2	Nil	Nil	Nil
Brand 3	Nil	Nil	Nil
Brand 4	4.0 × 10^4^	Nil	Nil
Brand 5	9.0 × 10^4^	Nil	Nil
Brand 6	Nil	Nil	Nil
Brand 7	Nil	Nil	Nil
Brand 8	2.3 × 10^4^	Nil	Nil
Brand 9	Nil	Nil	Nil
Brand 10	Nil	Nil	Nil

**Table:** (b) Microbial quality of sachet water collected from market site

Samples of sachet water	Total coliform/100 ml	Faecal coliform/100 ml	*Escherichia coli*
Market site	Market site	Market site	Market site
Brand 1	4.0 × 10^4^	Nil	Nil
Brand 2	Nil	Nil	Nil
Brand 3	Nil	Nil	Nil
Brand 4	4.0 × 10^4^	Nil	Nil
Brand 5	9.0 × 10^4^	Nil	Nil
Brand 6	Nil	Nil	Nil
Brand 7	Nil	Nil	Nil
Brand 8	2.3 × 10^4^	Nil	Nil
Brand 9	9.0 × 10^4^	Nil	Nil
Brand 10	Nil	Nil	Nil

**Table 7:** Physicochemical parameters of sachet water.

	Brand 6	Brand 9	Brand 7	Brand 1	Brand 5	Brand 6	Brand 8	Brand 2	Brand 3	Brand 3
_p_H	6.8	6.5	6.7	6.5	6.8	6.9	6.6	6.7	6.6	6.7
Colour	4	4	5	5	3	5	4	6	5	5
Alkaline	143	43	110	45	40	150	52	100	54	90
Hardness	125	39	90	75	80	60	90	120	96	120
Iron	0.07	0.01	0.05	0.02	0.1	0.09	0.08	0.1	0.1	0.08
Chloride	28	40	43	51	50	47	60	65	54	48

## Data Availability

The data used to support the findings of this study are available on request from the corresponding author.

## References

[1] Wardrop N. A., Dzodzomenyo M., Aryeetey G., Hill A. G., Bain R. E. S., Wright J. (2017). Estimation of packaged water consumption and associated plastic waste production from household budget surveys. *Environmental Research Letters*.

[2] de França Doria M., Pidgeon N., Hunter P. R. (2009). Perceptions of drinking water quality and risk and its effect on behaviour: a cross-national study. *Science of the Total Environment*.

[3] Fisher M. B., Williams A. R., Jalloh M. F., Saquee G., Bain R. E. S., Bartram J. K. (2015). Microbiological and chemical quality of packaged sachet water and household stored drinking water in Freetown, Sierra Leone. *PLoS One*.

[4] Adebayo O., Olugbenga E., Sunday E., Nchedo U. K. (2012). Microbiological examination of sachet water sold in Aba, Abia – State, Nigeria. *Global Research Journal of Microbiology*.

[5] Oludairo O., Aiyedun J. (2015). Contamination of commercially packaged sachet water and the public health implications: an overview. *Bangladesh Journal of Veterinary Medicine*.

[6] Oladipo I. C., Onyenike I. C., Adebiyi A. O. (2008). Microbiological analysis of sachet water vended in Ondo State, Nigeria. *Environmental Research Journal*.

[7] Addo K., Mensah G., Bekoe M., Bonsu C., Akyeh M. (2009). Bacteriological quality of sachet water produced and sold in Teshie-Nungua suburbs of Accra, Ghana. *African Journal of Food, Agriculture, Nutrition and Development*.

[8] Ahimah J. K., Ofosu S. A. (2012). Evaluation of the quality of sachet water vended in the New Juaben municipality of Ghana. *International Journal of Water Resources and Environmental Engineering*.

[9] Akinde S. B., Nwachukwu M. I., Ogamba A. S. (2011). Storage Effects on the Quality of Sachet Water Produced within Port Harcourt Metropolis , Nigeria. *Jordan Journal of Biological Sciences*.

[10] Mgbakor C., Ojiegbe G. C., Okonko I. O. (2011). Bacteriological evaluation of some sachet water on sales in Owerri metropolis, Imo State, Nigeria. *Malaysian Journal of Microbiology*.

[11] Fernández-Vázquez R., Hewson L., Fisk I. (2013). Colour influences sensory perception and liking of orange juice. *Flavour*.

[12] Wright J. A., Yang H., Rivett U., Gundry S. W. (2012). Public perception of drinking water safety in South Africa 2002–2009: a repeated cross-sectional study. *BMC Public Health*.

[13] Okechukwu R. I., Ogukwe C. E., Igboasoiyi O. O. (2015). Physico-chemical quality of different brands of sachet water sold in Federal University of Technology Campus Imo State, Nigeria. *Research Journal of Chemical Sciences*.

[14] Ugochukwu S., Giwa F. J., Giwa A. (2015). Bacteriological evaluation of sampled sachet water sold in Samaru-Zaria, Kaduna-State, Nigeria. *Nigerian Journal of Basic and Clinical Sciences*.

[15] Isikwue M. O., Chikezie A. (2014). Quality assessment of various sachet water brands marketed in Bauchi Metropolis of Nigeria. *International Journal of Advances in Engineering & Technology*.

[16] Stoler J., Tutu R. A., Ahmed H., Frimpong L. A., Bello M. (2014). Sachet water quality and brand reputation in two low-income urban communities in Greater Accra, Ghana. *American Journal of Tropical Medicine and Hygiene*.

[17] Dada A. (2009). Sachet water phenomenon in Nigeria : assessment of the potential health impacts. *African Journal of Microbiology Research*.

[18] Maduka H. C. C., Department of Biochemistry, Federal University of Technology, Owerri (FUTO), Imo State, Nigeria, Chukwu N. C. (2014). Assessment of commercial bottled table and sachet water commonly consumed in Federal University of Technology, Owerri (FUTO), Imo State, Nigeria using microbiological indices. *Journal of Dental and Medical Sciences*.

[19] Murcott S., Advisor T., Veneziano D. (2007). Water quality and business aspects of sachet-vended water in Tamale, Ghana.

[20] Ackah M., Anim A. K., Gyamfi E. T., Acquah J., Nyarko E. S., Kpattah L. (2012). Assessment of the quality of sachet water consumed in urban townships of Ghana using physico-chemical indicators : a preliminary study. *Advances in Applied Science Research*.

[21] Borketey P. B., Asmah R. H. (2007). Sachet drinking water in Accra : the potential threats of transmission of enteric pathogenic protozoan organisms. *Ghana Medical Journal*.

[22] Ngmekpele B. S., Hawkins J. (2014). Consumers’ perception of quality and health beliefs of sachet drinking water : evidence from Obuasi in the Ashanti region of Ghana. *Developing Country Studies*.

[23] Addo H. O., Addo K. K., Langbong B. (2014). Water handling and hygiene practices on the transmission of diarrhoeal diseases and soil transmitted helminthic infections in communities in rural Ghana. *Civil and Environmental Research*.

[24] Dzodzomenyo M., Fink G., Dotse-Gborgbortsi W. (2018). Sachet water quality and product registration: a cross-sectional study in Accra, Ghana. *Journal of Water and Health*.

